# Polysaccharide K suppresses angiogenesis in colon cancer cells

**DOI:** 10.3892/etm.2012.632

**Published:** 2012-07-04

**Authors:** YOSHIKI SATOH, TAKANORI GOI, TOSHIYUKI NAKAZAWA, YOUHEI KIMURA, YASUO HIRONO, KANJI KATAYAMA, AKIO YAMAGUCHI

**Affiliations:** First Department of Surgery, University of Fukui, Yoshida-gun, Fukui, Japan

**Keywords:** colon cancer, polysaccharide K, angiogenesis

## Abstract

The protein-bound polysaccharide K (PSK) is used as a non-specific immunotherapeutic agent for the treatment of colon cancer. Little research, however, has been conducted on its association with angiogenesis, which is a prognostic factor markedly correlated with hematogenous metastases. We therefore decided to investigate the action of PSK on angiogenic growth factors, angiogenesis inhibitors and angiogenesis in colon cancer cells. Reverse transcription-polymerase chain reaction (RT-PCR) was used to investigate changes in HIF-1α mRNA expression. PCR array was used to investigate changes in angiogenic growth factors and angiogenesis inhibitors, as well as the expression of related genes. Colon cancer cells were cultured with or without PSK for 48 h. The following day, cells were cultured for two days at 37°C in new complete media. The resulting culture medium was placed in the chamber of a tube formation system in order to investigate tube formation. Investigation of HIF-1α mRNA expression in colon cancer cell lines and in cells cultured under identical conditions with added PSK revealed a significant decrease in expression, as well as a decrease in angiogenic growth factors and related genes in PSK-treated colon cancer cell lines. By contrast, levels of angiogenesis inhibitors and related genes were higher in the PSK-treated colon cancer cell lines. Investigation of tube formation revealed that elongation was inhibited in the medium of the PSK-treated colon cancer cell lines in comparison to the medium of the non-treated colon cancer cell lines. PSK suppresses angiogenic growth factors and related genes, enhances angiogenesis inhibitors and related genes and ultimately suppresses angiogenesis in colon cancer cells.

## Introduction

Polysaccharide K (PSK; Kureha Chemical Industry Co., Ltd., Tokyo, Japan) is a protein-bound polysaccharide widely used as a non-specific immunotherapeutic agent and is derived from the cultured mycelia of *Coriolus versicolor*. This protein-polysaccharide complex, which has a molecular weight of approximately 940,000 Da, contains approximately 38% protein and a saccharide portion consisting of a glucan with approximately 75% glucose and smaller amounts of mannose, xylose and galactose (1). To date, PSK has been administered primarily to patients with gastric cancer, colon cancer and other gastrointestinal malignancies. Torisu *et al* reported that patients with curatively resected colon cancer had a significantly improved survival rate when treated with PSK (2). Yoshitani and Takashima (3) and Ohwada *et al* (4), who used PSK in combination with anticancer agents to treat curatively resected patients, also reported significantly improved survival in the patients who received PSK compared with those who did not.

The following main mechanisms of action of PSK on malignancies have been identified to date: i) direct apoptosis induction, inhibition of cellular infiltration and enhancement of MHC class-I expression; ii) enhancement of natural killer, cytotoxic T and lymphokine-activated killer activation and regulation of cytokine production; and iii) suppression of TGF-β production and reduction of oxidative stress (5–8). PSK also has a variety of immunostimulatory effects as a biochemical response modifier. Liver, lung and other hematogenous metastases are considered to be prognostic factors in colon cancer. Hematogenous metastases of colon cancer are generally believed to occur when cancer cells detach from the primary tumor, invade the capillaries and spread systemically via the portal and greater circulatory systems prior to adhering to vascular endothelial cells in the target organ, escaping and infiltrating outside blood vessels and proliferating (9,10). Previous characterization of the mechanisms of metastasis has identified key angiogenic growth factors in this process (11–13). Therefore, we investigated the changes induced by PSK in angiogenic growth factors, angiogenesis inhibitors and related genes in colon cancer cells, and whether PSK suppresses angiogenesis.

## Materials and methods

### Cell culture and PSK stimulation

Human colorectal cancer cell lines, SW620, HT29 and HCT116 (obtained from European collection of cell cultures, UK), were cultured at 37°C in 5% CO_2_ in RPMI-1640 medium containing 10% fetal bovine serum (14). Cells were seeded (5x10^5^) into 6-cm dishes in triplicate with PSK for 2 days.

### Cell viability

Apoptosis was detected by flow cytometry using Annexin V Detection kit (Nanjing KeyGen Biotech, Nanjing, China). Briefly, cells were double stained with Annexin V-TIRIC for 15 min at 37°C. After cells were washed thrice in PBS, we detected non-red cells under a fluorescent microscope.

### Reverse transcription-polymerase chain reaction (RT-PCR) analysis

The total RNA was extracted from the colorectal cancer cells using guanidinium-thiocyanate (15,16). Single strand cDNA was prepared from 3 μg of total RNA using Moloney murine leukemia virus reverse transcriptase (Takara Bio, Inc., Shiga, Japan). The primers for PCR amplification of the HIF-1α gene-coding regions were as follows: 5′ primer; HIF-1α -AX,GGACAAGTCACCACAGGA, 3′ primer; HIF-1α -BX,GGAGAAAATCAAGTCGTG. GAPDH amplification was used as an internal PCR control with 5′-GGGGAGCCAAAAGGGTCATCATCT-3′ as the sense primer and 5′-GACGCCTGCTTCACCACCTTCTTG-3′ as the antisense primer. A total of 23 cycles of denaturation (94°C, 1 min), annealing (50°C, 1.5 min) and extension (72°C, 2 min) were carried out in a thermal cycler (PTC-100, Programmable Thermal Controller, NJ Research Inc., MA, USA). The PCR products (10 μl) which demonstrated the relevant bands in RT-PCR analysis were sequenced by electrophoresis in 1.2% agarose gel. The sequencing was performed on PCR products that showed the bands in RT-PCR analysis.

### RT2 Profiler™ PCR array and real-time PCR

Total RNA was extracted from colon cancer cells using guanidiniumthiocyanate. Real-time PCR was performed according to the manufacturer’s instructions included with the RT2 Profiler PCR array system (angiogenic growth factors and angio-genesis inhibitors; PCR array: catalog no. PAHS-072A; SA Bioscience, Valencia, CA, USA). The data were analyzed using Excel-based PCR array data analysis templates.

### In vitro tube formation assay

Following preparation of the cells described above, the medium was removed from all dishes and replaced with fresh complete medium. After two days, each culture fluid was collected and added to wells of an angiogenesis kit (Kurabo Company, Japan). Fields from each sample were photographed and total tube length was analyzed by the MacSCOPE program (Mitani Company, Tokyo, Japan). The control tube areas were defined as 100% tube formation and the percent increase in tube formation as compared with the control was calculated for each sample (17).

### Statistical considerations

Other characteristics of the two treatment methods were compared using the Chi-square test. P<0.05 was considered to indicate a statistically significant result.

## Results

### Cell viability

The colon cancer cells analyzed under a fluorescence microscope using the Annexin-V assay demonstrated no increased cell apoptosis and death in samples treated with PSK (100 or 300 μg/ml) compared with untreated cells. Cells treated with 500 μg/ml demonstrated an increase in cell apoptosis and death (Table I).

### HIF-1α mRNA expression with PSK exposure in colon cancer cell lines

**Figure 1:**
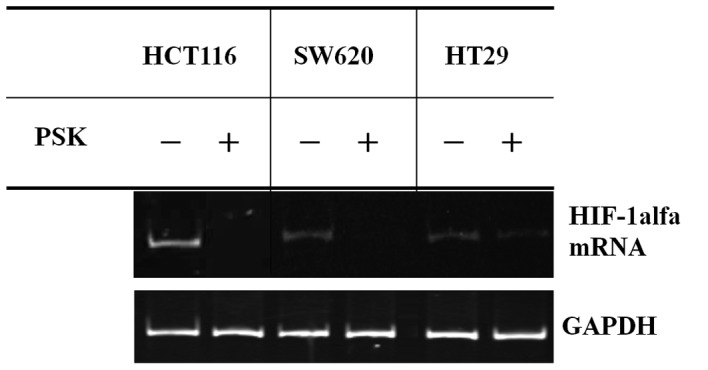
The expression of HIF-1α mRNA was detected in colon cancer cell lines. The HIF-1α mRNA expression in colon cancer cell lines treated with PSK was decreased. PSK, polysaccharide K.

RT-PCR was used to investigate HIF-1α mRNA expression in colon cancer cell lines. The results are shown in Fig. 1. Although the expression of HIF-1α mRNA was detected in colon cancer cell lines, the addition of PSK suppressed HIF-1α mRNA expression in colon cancer cell lines.

### Expression of angiogenic growth factors in colon cancer cell lines treated with PSK

PCR array was used to investigate how the addition of PSK to colon cancer cell lines affected levels of angiogenic growth factors and related genes. A comparison of levels in these cells to those in untreated colon cancer cell lines cultured is listed in Table II. Typical genes that were expressed at lower levels included gastrin-releasing peptide (GRP), interleukin 8 (IL8) and platelet-derived growth factor β polypeptide (PDGFB) in HCT116, EGF-like repeats and discoidin I-like domains 3 (EDIL3) in SW620 and chemokine (C-X-C motif) ligand 9 (CXCL9), fibroblast growth factor binding protein 1 (FGFBP1) and interleukin 8 (IL8) in the HT29 cell line. Numerous other angiogenic growth factors and the expression of related genes were reduced in all cell types.

### Expression of angiogenesis inhibitors in colon cancer cell lines treated with PSK

PCR array was used to investigate how the addition of PSK to colon cancer cell lines affected levels of angiogenesis inhibitors and related genes. A comparison of levels in these cells to those in untreated colon cancer cell lines cultured at 20% CO2 is listed in Table III. Typical genes that were expressed at higher levels included TIMP metallopeptidase inhibitor (TIMP1) in HCT116 and interleukin 12A (IL12A) and troponin I type 3 (TNNI3) in the HT29 cell line. There were no typical genes with an altered expression pattern in the SW620 cell line.

### Tube formation in colon cancer cell lines treated with or without PSK

**Figure 2:**
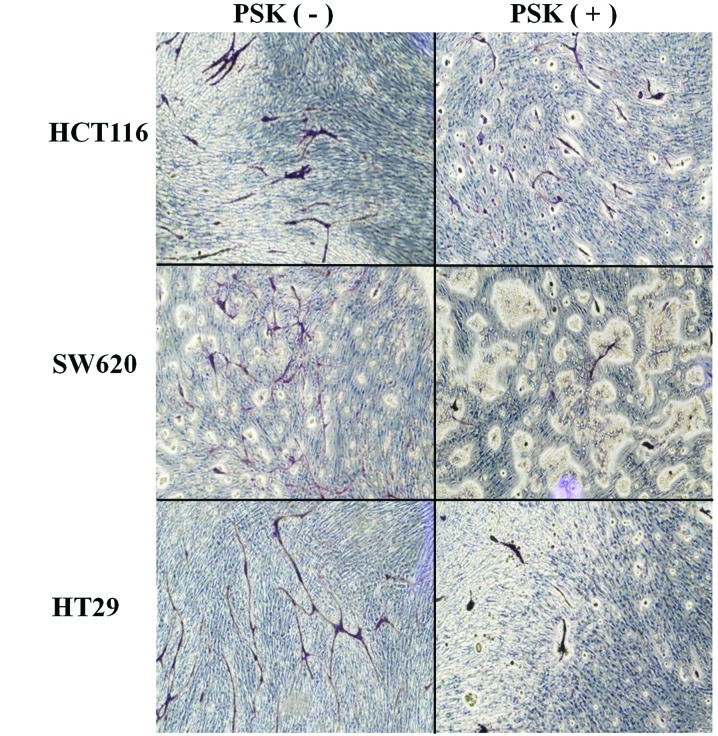
Tube formation in PSK-stimulated colon cancer cells. PSK-treated or untreated colon cancer cell lines were applied to the wells of a tube formation assay to investigate the effects on elongation of tube formation. The length was significantly decreased in PSK-stimulated colon cancer cells compared with untreated cells. PSK, polysaccharide K.

**Figure 3:**
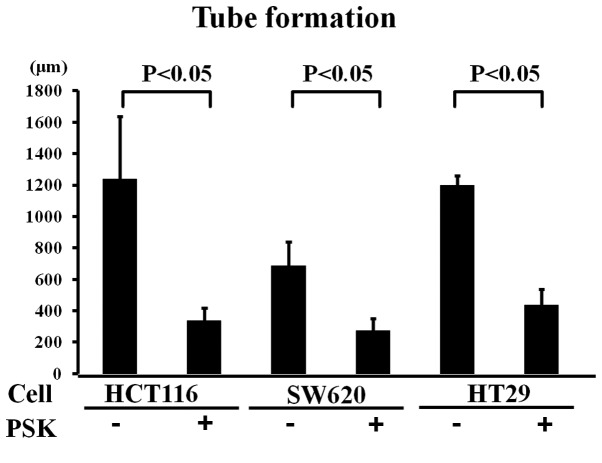
Evaluation of the tube formation in PSK-stimulated colon cancer cells. With tube elongation in the medium of untreated colon cancer cell lines taken to be 100%, the elongation of the PSK-treated cell lines was 40% in SW620, 27% in HCT116 and 36.5% in HT29. PSK, polysaccharide K.

The medium from PSK-treated colon cancer cell lines was applied to the wells of a tube formation assay to investigate the effects of PSK on the elongation of tube formation. Tube elongation in the medium of untreated colon cancer cell lines was taken to be 100%, elongation was 40% in SW620, 27% in HCT116 and 36.5% in HT29 cells cultured in the medium of PSK-treated colon cancer cell lines (Figs. 2 and 3). Elongation was therefore significantly less than that observed in the medium of non-treated colon cancer cell lines.

## Discussion

PSK, derived from the cultured mycelia of *C. versicolor*, is widely used as a nonspecific immunotherapeutic agent (1,5–8). The efficacy of PSK has been demonstrated to increase survival in patients with gastrointestinal malignancies, including gastric and colon cancer. Hematogenous metastases are considered to be a prognostic factor in colon cancer, and PSK is believed to act in the process leading to these metastases, thereby increasing survival (2–4). It has been reported that the occurrence of hematogenous metastases in colon cancer is closely correlated with increased angiogenesis, and angiogenic growth factors and angiogenic growth inhibiting factors likely contribute to the induction and propagation of angiogenesis and may eventually promote hematogenous metastases (9–13).

We investigated how the addition of PSK to the medium of cultured colon cancer cell lines affects the expression of the HIF-1α gene, which is closely associated with the expression of angiogenic growth factors, in addition to angiogenic growth factors and angiogenesis (18–23).

The expression of HIF-1α mRNA was detected in colon cancer cell lines, but the addition of PSK suppressed HIF-1α mRNA expression. The HIF-1α gene is believed to activate the production of numerous angiogenic growth factors, and has various effects on cancer, regulating at least 70 genes, most of which promote cancer (18–23). Also HIF-1α gene, oncogene and tumor suppressor gene intricately linked with the expression of angiogenic growth factors and angiogenesis inhibitors (24). A PCR array was then used to investigate the affected angiogenic growth factors and angiogenesis inhibitors. Although the suppression of genes differed between the cell lines studied, the addition of PSK suppressed numerous angiogenic growth factors and increased levels of angiogenesis inhibitors.

When the untreated colon cancer cell lines were used in a tube formation system, tube formation was promoted. By contrast, when the PSK-treated colon cancer cell lines were used, tube formation was reduced, which indicates that PSK acts to suppress angiogenesis in the strains of colon cancer cells studied.

The effects of PSK identified in the present study include the suppression of HIF-1α gene expression, the suppression of angiogenic growth factors and the enhancement of angiogenesis inhibitors in colon cancer cells. These findings demonstrate the potential of PSK to ultimately suppress angiogenesis.

## Figures and Tables

**Table I:** Cell viability following exposure to PSK.

PSK (μg/ml)	Annexin V staining (%)
0	3.2
100	3.5
300	3.8
500	10.0

PSK, polysaccharide K.

**Table II:** Representative list of downregulated genes in PSK-stimulated cells (angiogenic growth factors and related genes).

Cell line	Gene Bank	Description	Ratio
HCT116	Hs.153444	GRP, gastrin-releasing peptide	−5.2635
	Hs.624	IL8, interleukin 8	−4.0425
	Hs.1976	PDGFB, platelet-derived growth factor β polypeptide	−4.9113
SW620	Hs.482730	EDIL3, EGF-like repeats and discoidin I-like domains 3	−11.0357
HT29	Hs.77367	CXCL9, chemokine (C-X-C motif) ligand 9	−28.9895
	Hs.1690	FGFBP1, fibroblast growth factor binding protein 1	−4.4097
	Hs.624	IL8, interleukin 8	−19.315

PSK, polysaccharide K.

**Table III:** Representative list of upregulated genes in PSK-stimulated cells (angiogenesis inhibitors and related genes).

Cell line	Gene Bank	Description	Ratio
HCT116	Hs.522632	TIMP1, TIMP metallopeptidase inhibitor 1	5.7541
SW620	-	-	-
HT29	Hs.673	IL12A, interleukin 12A (natural killer cell stimulatory factor 1, cytotoxic lymphocyte maturation factor 1, p35)	17.1
	Hs.644596	TNNI3, troponin I type 3 (cardiac)	4.1713

PSK, polysaccharide K.

## References

[1] Tsukagoshi S, Hashimoto Y, Fujii G, Kobayashi H, Nomoto K, Orita K (1984). Krestin (PSK). Cancer Treat Rev.

[2] Torisu M, Hayashi Y, Ishimitsu T, Fujimura T, Iwasaki K, Katano M, Yamamoto H, Kimura Y, Takesue M, Kondo M, Nomoto K (1990). Significant prolongation of disease-free period gained by oral polysaccharide K (PSK) administration after curative surgical operation of colorectal cancer. Cancer Immunol Immunother.

[3] Yoshitani S, Takashima S (2009). Efficacy of postoperative UFT (Tegafur/Uracil) plus PSK therapies in elderly patients with resected colorectal cancer. Cancer Biother Radiopharm.

[4] Ohwada S, Ikeya T, Yokomori T, Kusaba T, Roppongi T, Takahashi T, Nakamura S, Kakinuma S, Iwazaki S, Ishikawa H (2004). Adjuvant immunochemotherapy with oral Tegafur/Uracil plus PSK in patients with stage II or III colorectal cancer: a randomized controlled study. Br J Cancer.

[5] Araya S, Nio Y, Hayashi H, Masai Y, Tsubono M, Ishigami S, Imamura M (1994). Various plant-derived polysaccharides augment the expression of HLA on Colo205 human colonic cancer line. J Jpn Soc Cancer Ther.

[6] Hirose K, Zachariae CO, Oppenheim JJ, Matsushima K (1990). Induction of gene expression and production of immunomodulating cytokines by PSK in human peripheral blood mononuclear cells. Lymphokine Res.

[7] Algarra I, Collado A, Garcia Lora A, Garrido F (1999). Differential effect of protein-bound polysaccharide (PSK) on survival of experimental murine tumors. J Exp Clin Cancer Res.

[8] Harada M, Matsunaga K, Oguchi Y, Iijima H, Tamada K, Abe K, Takenoyama M, Ito O, Kimura G, Nomoto K (1997). Oral administration of PSK can improve the impaired anti-tumor CD4^+^ T-cell response in gut-associated lymphoid tissue (GALT) of specific-pathogen-free mice. Int J Cancer.

[9] Fidler IJ, Ellis LM (1994). The implications of angiogenesis for the biology and therapy of cancer metastasis. Cell.

[10] Hanahan D, Folkman J (1996). Patterns and emerging mechanisms of the angiogenic switch during tumorigenesis. Cell.

[11] Stoeltzing O, Liu W, Reinmuth N, Parikh A, Ahmad SA, Jung YD, Fan F, Ellis LM (2003). Angiogenesis and antiangiogenic therapy of colon cancer liver metastasis. Ann Surg Oncol.

[12] Ishigami SI, Arii S, Furutani M, Niwano M, Harada T, Mizumoto M, Mori A, Onodera H, Imamura M (1998). Predictive value of vascular endothelial growth factor (VEGF) in metastasis and prognosis of human colorectal cancer. Br J Cancer.

[13] Tokunaga T, Oshika Y, Abe Y, Ozeki Y, Sadahiro S, Kijima H, Tsuchida T, Yamazaki H, Ueyama Y, Tamaoki N, Nakamura M (1998). Vascular endothelial growth factor (VEGF) mRNA isoform expression pattern is correlated with liver metastasis and poor prognosis in colon cancer. Br J Cancer.

[14] Goi T, Yamaguchi A, Nakagawara G, Urano T, Shiku H, Furukawa K (1998). Reduced expression of deleted colorectal carcinoma (DCC) protein in established colon cancers. Br J Cancer.

[15] Fujishima Y, Goi T, Kimura Y, Hirono Y, Katayama K, Yamaguchi A (2012). MUC2 protein expression status is useful in assessing the effects of hyperthermic intraperitoneal chemotherapy for peritoneal dissemination of colon cancer. Int J Oncol.

[16] Goi T, Fujioka M, Satoh Y, Tabata S, Koneri K, Nagano N, Hirono Y, Katayama K, Hirose K, Yamaguchi A (2004). Angiogenesis and tumor proliferation/metastasis of human colorectal cancer cell line SW620 transfected with endocrine glands-derived-vascular endothelial growth factor, as a new angiogenic factor. Cancer Res.

[17] Nagano H, Goi T, Koneri K, Hirono Y, Katayama K, Yamaguchi A (2007). Endocrine gland-derived vascular endothelial growth factor (EG-VEGF) expression in colorectal cancer. J Surg Oncol.

[18] Semenza GL (2010). Oxygen homeostasis. Wiley Interdiscip Rev Syst Biol Med.

[19] Semenza GL (2009). HIF-1 inhibitors for cancer therapy: from gene expression to drug discovery. Curr Pharm Des.

[20] Liao D, Johnson RS (2007). Hypoxia: a key regulator of angiogenesis in cancer. Cancer Metastasis Rev.

[21] Chan DA, Giaccia AJ (2007). Hypoxia, gene expression, and metastasis. Cancer Metastasis Rev.

[22] Zhou J, Schmid T, Schnitzer S, Brüne B (2006). Tumor hypoxia and cancer progression. Cancer Lett.

[23] Harris AL (2002). Hypoxia – a key regulatory factor in tumour growth. Nat Rev Cancer.

[24] Schmid T, Zhou J, Köhl R, Brüne B (2004). p300 relieves p53-evoked transcriptional repression of hypoxia-inducible factor-1 (HIF-1). Biochem J.

